# Mitochondrial FAD-linked Glycerol-3-phosphate Dehydrogenase: A Target for Cancer Therapeutics

**DOI:** 10.3390/ph7020192

**Published:** 2014-02-11

**Authors:** Gurmit Singh

**Affiliations:** Department of Pathology and Molecular Medicine, McMaster University, 1280 Main Street West, Hamilton, Ontario L8S 4K1, Canada; E-Mail: singhg@mcmaster.ca; Tel.: +1-905-525-9140 (ext. 28144); Fax: +1-905-777-7856

**Keywords:** drug discovery, glycerol-3-phosphate dehydrogenase, high-throughput screening, hydrogen peroxide, reactive oxygen species, prostate cancer

## Abstract

Imbalances in cellular redox state are frequently observed in cancer cells, and contribute significantly to cancer progression and apoptotic resistance. Hydrogen peroxide (H_2_O_2_) is one reactive oxygen species (ROS) that is produced in excess within cancer cells. In this study, we investigated the mitochondrial glycerol-3-phosphate-dependent (GPD2) ROS production in PC-3 cells and demonstrated the importance of excessive H_2_O_2_ production on their survival. By exploiting the abnormal H_2_O_2_ production of PC-3 cells, we initiated a high-throughput screening of the *Canadian Compound Collection*, composed of 29,586 small molecules, targeting the glycerophosphate-dependent H_2_O_2_ formation in PC-3 cells. Eighteen compounds were identified to have significant inhibitory activity. These compounds have not been previously characterized as inhibitors of the enzyme. Six of these compounds were further analyzed in PC-3 cells and dose response studies displayed an inhibitory and anti-oxidative potency that ranged from 1 µM to 30 µM. The results presented here demonstrate that inhibitors of mitochondrial GPD2 activity elicit anti-proliferative effects on cancer cells.

## 1. Introduction

Most cancer types display elevated levels of reactive oxygen species (ROS) [1]. Prostate cancer is characterized by innate oxidative stress, which is a hallmark of the aggressive phenotype of this disease [2]. Both healthy and malignant cells utilize hydrogen peroxide (H_2_O_2_), a stable and diffusible form of ROS, as an effective signaling molecule in cellular mitogenic pathways [3,4,5]. However, prostate cancer cells exhibit abnormally increased production of H_2_O_2_, which influences many aspects of the disease, including proliferation, survival, and metastasis [2,6]. Mitochondria are recognized as a predominant source of ROS in both healthy and malignant cells, and within them, at least nine ROS-generating enzymes are known [7]. One mitochondrial enzyme, flavin-linked glycerol-3-phosphate ubiquinone oxidoreductase, also known as mitochondrial glycerol-3-phosphate dehydrogenase (GPD2) [EC 1.1.5.3], is capable of H_2_O_2_ production [8,9,10,11]. GPD2-dependent H_2_O_2_ formation has been reported in insect and mammalian mitochondria [9,10,11]. In isolated *Drosophila* mitochondria, 70% of the total cellular H_2_O_2_ production was estimated to stem from GPD2 [10]. GPD2 emerged as a substantial source of H_2_O_2_ in the pro-oxidative environment of the prostate cancer cell lines PC-3, LNCaP, DU145, and CL1 [8]. In particular, PC-3 cells, which are derived from aggressive metastatic adenocarcinoma [12], displayed the highest GPD2 activity among the examined prostate cancer cell lines, generating up to 4-fold higher amounts of H_2_O_2_ compared to normal prostate epithelial cells [8].

GPD2 and the cytoplasmically-localized NAD-linked glycerol-3-phosphate dehydrogenase (GPD1) [EC 1.1.1.8] are the two components of the glycerophosphate shuttle, which, alongside the malate-aspartate transporter, build a secondary, rapidly operating biochemical mechanism utilized for the reoxidation of glycolytic-formed NADH. GPD2, localized on the outer surface of the inner mitochondrial membrane [13], unidirectionally channels the glycerol-3-phosphate-derived electrons in the mitochondrial inner membrane. In the pro-oxidative mitochondrial environment, GPD2 contributes to superoxide anion formation (O_2_^−^), which further dismutates primarily via superoxide-dismutases to form H_2_O_2_. The molecular mechanism of GPD2-dependent ROS production has not yet been determined, although a recent report suggests that coenzyme Q may be the site of ROS generation [14].

Metabolically, GPD2 participates in glycolysis, gluconeogenesis, glycerol and lipid metabolism (triacylglycerol metabolism) [15]. The GPD2-dependent glycerol-3-phosphate oxidation and the glycerol-phosphate shuttle mechanism are implicated in the process of thermogenesis [16,17]. GPD2 is reported to exhibit a pathophysiological relationship to type II diabetes [18,19], possibly due to the involvement in the maintenance of the pancreatic β-cell redox status [20,21]. Impaired GPD2 activity was reported in type II diabetic patients [20] and mutations of the GPD2 gene were documented in families of patients with non-insulin-dependent diabetes [22]. Also, antibodies against GPD2 were detected in a large number of diabetic patients [23]. Furthermore, functional GPD2 defects have also been suggested in mild non-syndrome mental retardation [24,25]. Finally, various animal models of oxidative stress support the notion that mitochondrial-generated ROS may have a causal role in atherosclerosis and other cardiovascular diseases [26].

The role of GPD2 in producing ROS is a poorly investigated metabolic side-reaction with profound implications for the proliferation of prostate cancer cells. Consequently, we initiated an approach for the development of GPD2 inhibitors as potential cancer therapeutic agents. To exploit the abnormal H_2_O_2_ production of PC-3 cells [2,8], we propose a high-throughput screen (HTS) of the *Canadian Compound Collection* (CCC), composed of 29,586 small molecules, to target GPD2-dependent H_2_O_2_ formation in PC-3 cells. Active compounds will be further analyzed to determine their ROS-inhibiting parameters and the impact of H_2_O_2_ alteration on cancer cell proliferation.

## 2. Experimental

### 2.1. Cell Lines and Growth Conditions

The PC-3 prostate cancer cells [12] served as a target for ROS inhibition. They were grown in 10 cm tissue culture dishes containing RPMI 1640 media (Invitrogen, Burlington, ON, Canada) supplemented with 10% fetal bovine serum (FBS), 1% 100 mM sodium pyruvate, 1% antibiotic/antimycotic, and 1% 1M HEPES. Cells were incubated at 37°C and 5% CO_2_ in a humidified environment. PNT1A cells [27] resembled the normal epithelial cell control, and were similarly cultured in RPMI 1640 media, with 10% FBS and 1% antibiotic/antimycotic, and incubated as above.

### 2.2. Screening Library

The library screened at McMaster University’s High Throughput Screening (HTS) laboratory consisted of 29,586 compounds arrayed in 352 (96-well) plates as single compounds at 250 mM in 5% DMSO. The quality of all compounds in the CCC was assured by the vendors and the HTS laboratory to be greater than 95% pure. The library was screened at a constant 1:25 dilution with a 10 mM final concentration of compounds in each well (yielding 0.2% DMSO as final vehicle concentration). The library was screened over a 12-week period. For secondary screening, compounds 5108184, 5228270, 5477644, and 5543723 were purchased from ChemBridge (San Diego, CA, USA), and compounds RH02211 and KM04416 we purchased from Fisher Scientific Company (Ottawa, ON, Canada).

### 2.3. High-Throughput Screening for Small Molecules Altering H_2_O_2_ Production

In preparation for screening, cells were washed with 10 mL PBS, and trypsinized using 2 mL (1×) trypsin-EDTA solution in phosphate-buffered saline (PBS) for 5 min at 37 °C. Trypsinized cells from 3 tissue culture dishes were re-suspended in supplemented media (8 mL/plate) and transferred to a single sterile conical tube. Using a disposable hemacytometer (CELL-VU^®^, Fisher Scientific, Pittsburgh, PA, USA), pooled cells from the conical tube were counted under an inverted microscope. A solution containing 5,000 cells/100 µL were transferred to a robotic reservoir using a BIOMEK FX liquid handler (Beckman Coulter Inc., Fullerton, CA, USA) equipped with a 96-channel head. These were transferred to 92 wells of 96-well clear tissue-culture treated microplates. The remaining 4 wells of the microplate received media alone (no cells). Plates were then incubated for 24 h, after which the automated liquid handler removed the media, washed the wells once with PBS, and dispensed all compounds and controls.

After compound addition, the assay plates were incubated at room temperature for 15 min before performing the H_2_O_2_ detection assay. Extracellular H_2_O_2_ was assessed using Amplex^®^ Red reagent (10-acetyl-3,7-dihydroxyphenoxazine, Invitrogen) which, in combination with HRP, detects H_2_O_2_ released from PC-3 cells to produce the red fluorescent oxidation product resorufin, quantifiable fluorometrically [28]. Each compound plate was tested in duplicate. 100 µL of the Amplex^®^ Red reagent/horseradish peroxidase (HRP) working solution in PBS (25 µM Amplex^®^ Red in DMSO, 0.1 U/mL HRP in PBS) were added to each well using a second automated liquid handler. The reaction was allowed to proceed at room temperature, protected from light, for a minimum of 1 h and a maximum of 3.5 h. The cells were measured fluorometrically using the EnVision 2102 multilabel reader (Perkin Elmer, Waltham, MA, USA) in the continuous assay mode. The fluorescence was measured at 590 nm every 40 min to follow the kinetics of the reaction, using excitation and emission filters of 535 nm (25 nm bandwidth) and 600 nm (8 nm bandwidth), respectively.

PC-3 cells with vehicle only (0.2% DMSO) were used as screening positive controls. Medium only (with 0.2% DMSO) was found to be the best negative control for this assay during HTS. Use of 10 mM FCCP, an ionophore [29] that reduces PC-3 cell H_2_O_2_ formation by up to 30%, served as an intraplate reference control for the assay.

### 2.4. Assay Optimization and Validation for High-Throughput Screening

The Z′-factor [30] for the assay was calculated to assess the variability of the method and the bandwidth between positive and negative controls according to the following equation, where 𝜎 = standard deviation of positive (*p*) and negative (*n*) controls, µ = mean of positive (*p*) and negative (*n*) controls:

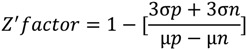
(1)


The wells containing the positive and negative controls were used to determine the relative activities per plate and to calculate the activity values for each well containing the compounds. The Z′-factor for each plate was calculated to assess the intra-plate variability. The fluorescence unit data obtained from each well was calculated in absolute percentage of residual activity. The residual activities of the plate and of the replica plate were plotted against each other and the hit zone estimated as the total average minus the standard deviation.

### 2.5. Identification of GPD2-Specific Inhibitors

To identify selective inhibitors of GPD2-related H_2_O_2_ production, GPD2 enzyme was purified from PC-3 cell cDNA. A *GPD2* construct (aa 43-727) was generated with an N-terminal Strep tagII sequence. Proteins were expressed in the bacterial overexpressing strain GJ1158 [31] and purified by strep-Tactin affinity chromatography. Individual samples of compounds were re-arrayed in 96-well plates and tested for GPD2 oxidoreductase activity. Recombinant GPD2 enzyme was added reactions were initiated with 30 mM glycerol-3-phosphate and assayed by the reduction of resazurin (Sigma-Aldrich, Oakville, ON, Canada), an artificial electron acceptor. The plates were measured using the Cytofluor Series 4000 Fluorescence Plate Reader (Excitation/Emission = 530 nm/590 nm; Perseptive Biosystems, Framingham, MA, USA).

### 2.6. Determination of EC_50_ Values

For compounds in the hit zone of the HTS assay (±3 standard deviations) and showing GPD2 oxidoreductase inhibitory activity, follow-up analyses were performed. The 50% effective concentration (EC_50_) for decreasing H_2_O_2_ production activity was assessed with Amplex^®^ Red. The compounds were added at concentrations varying from 1 × 10^−5^ to 0.2 M, and assays were conducted in quadruplicate.

### 2.7. Proliferation Assay

In proliferation assays extending over a period of 72 h, cells were initially seeded in 96-well plates at 5,000 cells/well in RPMI 1640 supplemented media. Cells were left undisturbed for 4 h prior to compound treatment. Compounds were dissolved in sterile PBS and added to the wells at the required concentrations. Bovine catalase (Sigma) was dissolved in PBS and sterilized by passage through 0.2 µm filter. Following 72 h of growth, in the presence or absence of catalase or compound, colonies were formalin fixed, stained with crystal violet, washed, dried, developed with a 50% ethanol PBS mixture, and optical density was measured at 570 nm.

### 2.8. Cytotoxicity Assays

The cytotoxic percentage was determined by measuring lactate dehydrogenase release 24 h after addition of the small molecule compound using the CytoTox 96 non-radioactive cytotoxicity assay kit (Promega, Madison, WI, USA) following instructions provided by the manufacturer. Cells were seeded at 5,000 cells/well in the presence or absence of the compound. Metabolic activity of cells was also assessed fluorometrically using Alamar Blue reduction according to the manufacturer’s instructions (Invitrogen) and trypan blue staining using hemocytometer counting to assess cell viability in the presence of inhibitors.

### 2.9. Statistical Analysis

All results were calculated from a minimum of three independent experiments and with replicates per experiment as indicated. Results were considered significant at *p* < 0.05 as calculated by one-way single-factor ANOVA with Tukey’s post-tests. Data analyses were conducted using GraphPad Prism (La Jolla, CA, USA).

## 3. Results and Discussion

GPD2 is a nuclear-encoded protein located at the outer surface of the inner mitochondrial membrane [13] and participates in lipolysis, gluconeogenesis and glycolysis [32]. We previously demonstrated that GPD2 activity is elevated in several human prostate cancer cell lines when compared with normal prostate epithelial cells [8,33]. GPD2 is a potent generator of ROS in prostate cancer cells and hence serves a pivotal role in the cancer’s pro-oxidative phenotype [8,33]. Limiting the H_2_O_2_ stress in PC-3 cells affected their growth, indicating that elevated H_2_O_2_ production is necessary for the survival of this malignant cell type. As glycerophosphate-dependent H_2_O_2_ production is a significant source of ROS in PC-3 cells, we analyzed its potential as a drug-sensitive target in the current investigation. To date, GPD2 has not been considered a target for HTS due to its challenging physico-chemical properties [34]. In particular, its sub-cellular localization [13] and the difficulty in generating sufficient quantities of purified [35,36], functional protein have limited its accessibility to small molecule screening approaches. However, knowing the GPD2-dependent pathophysiological characteristics of PC-3 cells, we addressed this issue by employing a whole-cell based HTS utilizing the prostate cancer cell line PC-3.

### 3.1. High-Throughput Screening for the iSolation of Compounds Altering H_2_O_2_ Production

**Figure 1:**
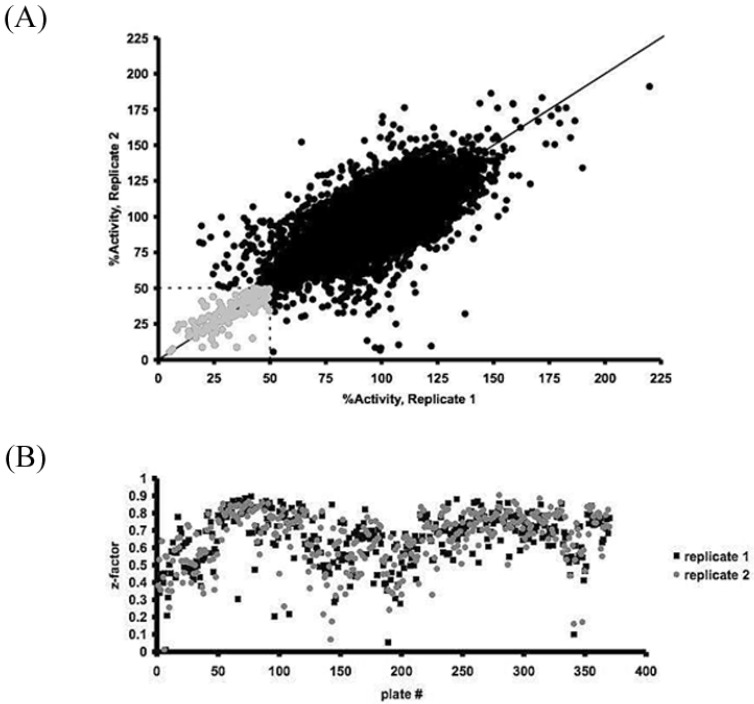
High-throughput screening of the *Canadian Compound Collection* for inhibitors of ROS formation in the PC-3 prostate cancer cell line. (**A**) Plot of the two screening replicates reported as percent residual activity relative to the average of the high controls. Hits were identified as molecules reducing the H_2_O_2_ activity to less than 50% in both replicates, a statistical cut-off three standard deviations below the high control mean. (**B**) Plot of the Z-factor values of the two replicates *versus* the plate number that demonstrates the robustness of the high-throughput screening assay.

A cell-based high-throughput screening protocol successfully identified cell-permeable effector molecules actively altering H_2_O_2_ formation in PC-3 cells. Active compounds were defined as those reducing H_2_O_2_ production activity to a level of at least three standard deviations below or above the high control in both replicates (average high control = 98.4%, 𝜎 = 14.35). A total of 133 compounds were identified as hits (Figure 1), 9 of which have been discontinued by the supplier. Additionally, 20 compounds *increased* H_2_O_2_ production activity significantly. The identified molecules are chemically diverse and do not fit into one particular class of compounds. The Z′-factor for the HTS assay for H_2_O_2_ production was determined to be 0.66 ± 0.13, indicating an excellent assay and ensuring an adequate signal-to-noise ratio.

Among the identified molecules, several natural compounds with known antioxidative and anti-proliferative activities were found to inhibit H_2_O_2_ formation in PC-3 cells (carnosic acid, caffeic acid, rottlerin, gingerol, hypericin). A number of molecules act via G protein-coupled cell receptors (dopamine, H5T3, serotonin, glutamate, *etc.*), indicating interconnecting points between signaling cascades and the cellular ROS generation machinery. Molecules such as calmidazolium, a reported calmodulin antagonist, specify a role of intracellular Ca^2+^ levels in the ROS production. Mercurial molecules such as thimerosal and phenylmercuric acetate suggest the involvement of thiol residues in H_2_O_2_ formation.

### 3.2. GPD2 Specificity

**Figure 2:**
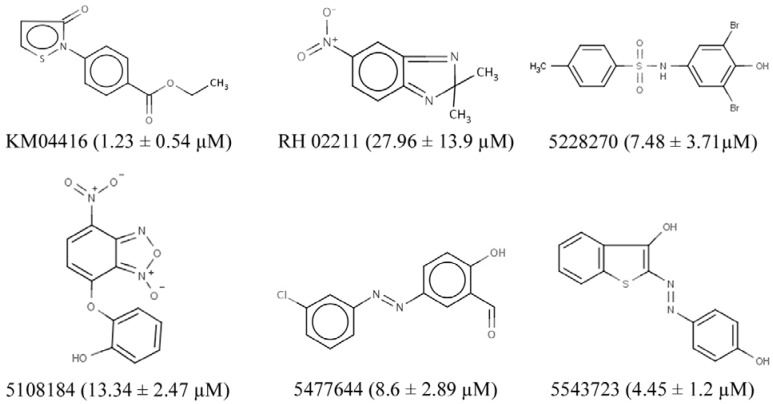
GPD2 activity inhibitors identified using HTS. The chemical structures of the six GPD2 activity inhibitors selected for follow-up analysis are shown, as well as their calculated EC_50_ values ± SEM.

**Figure 3:**
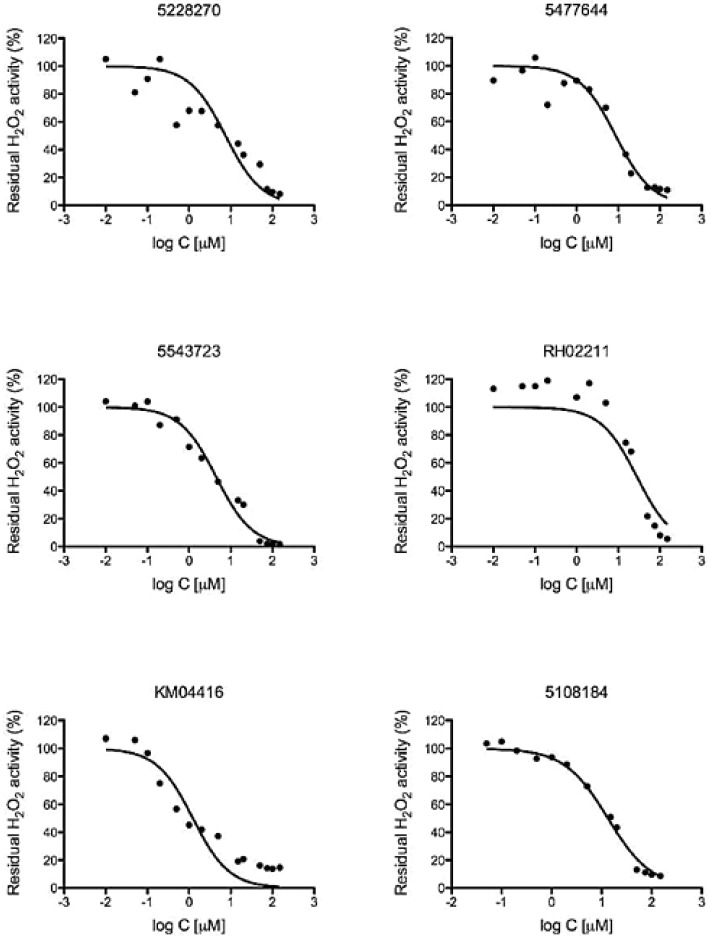
H_2_O_2_ activity inhibition curves for the most potent small molecule GPD2 inhibitors. Each curve represents the mean ± SEM of three independent experiments with six wells per experiment. The curve fit was calculated using a non-linear fit log (C_inhibitor_) *versus* normalized response.

Eighteen small molecule compounds from the inhibitors and one from the activators were identified as inhibiting the oxidoreductase activity of GPD2 to a level below 50% residual activity. From the known natural compounds of the CCC, lasalocid acid, caffeic acid, caffeic acid phenetyl ether (CAPE), rottlerin, carnosic acid, calmidazolium, propylnorapomorphine, SB 415286, and rhodomyrtoxin B emerged as effective GPD2 inhibitors, but were not selected for further screening in the current study as they had been examined by other groups. GPD2 activity was markedly inhibited by thiol-modifying mercurial reagents, such as thimerosal and phenylmercuric acetate, suggesting a physiological role for cysteine residues in GPD2’s function. Six compounds from the ChemBridge and Maybridge compound sub-libraries that had not been examined in previous studies were identified and further assessed for oxidoreductase activity (Figure 2). The six selected compounds were further analyzed for H_2_O_2_ inhibitory effects (Figure 3) and all maintained an anti-oxidative potency that ranged from 1 µM to 30 µM.

### 3.3. H_2_O_2_ is Required for PC-3 Cell Growth

**Figure 4:**
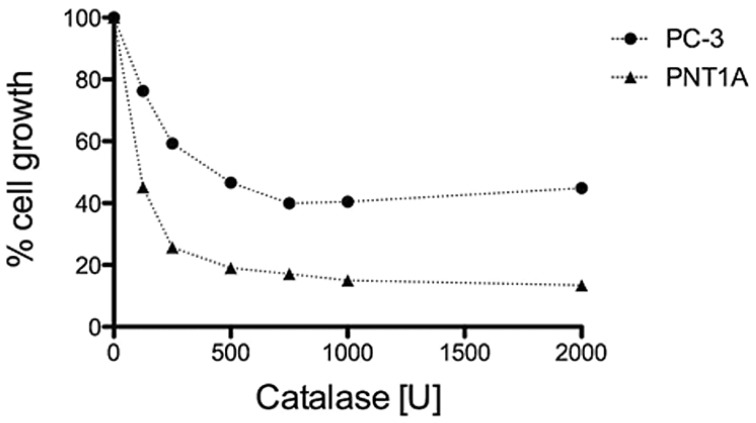
Growth effects of H_2_O_2_ elimination on PC-3 and PNT1A cells. Cell numbers were measured using crystal violet following a 72 h continuous exposure, and are displayed as % cell growth compared to untreated cells. The displayed data represent the average ±SEM of three independent experiments. Dose-dependent growth inhibition by catalase was observed in both cell lines, with PC-3 cells requiring higher concentration of catalase (IC_50_ = 580 ± 160 U/mL) to block cell proliferation, and PNT1A cells requiring lower concentration of catalase (IC_50_ = 75 ± 40 U/mL). The IC_50_ values were calculated using curve fit (GraphPad Prism) and applying a non-linear fit log (C_inhibitor_) *versus* normalized response.

PC-3 cells constitutively produce high amounts of H_2_O_2_ that diffuses into the extracellular space [8]. We evaluated the importance of this abnormal PC-3 characteristic for cell survival through the addition of sterile bovine catalase to the culture media [37]. The growth of PC-3 cells was inhibited in a dose-dependent manner; exclusion of this treatment by incubation or heat inactivation of the enzyme reversed this effect (Figure 4). Comparison with normal prostate epithelial cells PNT1A [27] the evaluation of the 50% inhibitory concentration of catalase confirmed that PC-3 cells produce significantly higher amounts of H_2_O_2_ (IC_50_ = 580 U/mL) compared to PNT1A (IC_50_ = 75 U/mL).

### 3.4. GPD2 Activity Inhibitors Impact Survival of Prostate Cancer Cells

**Figure 5:**
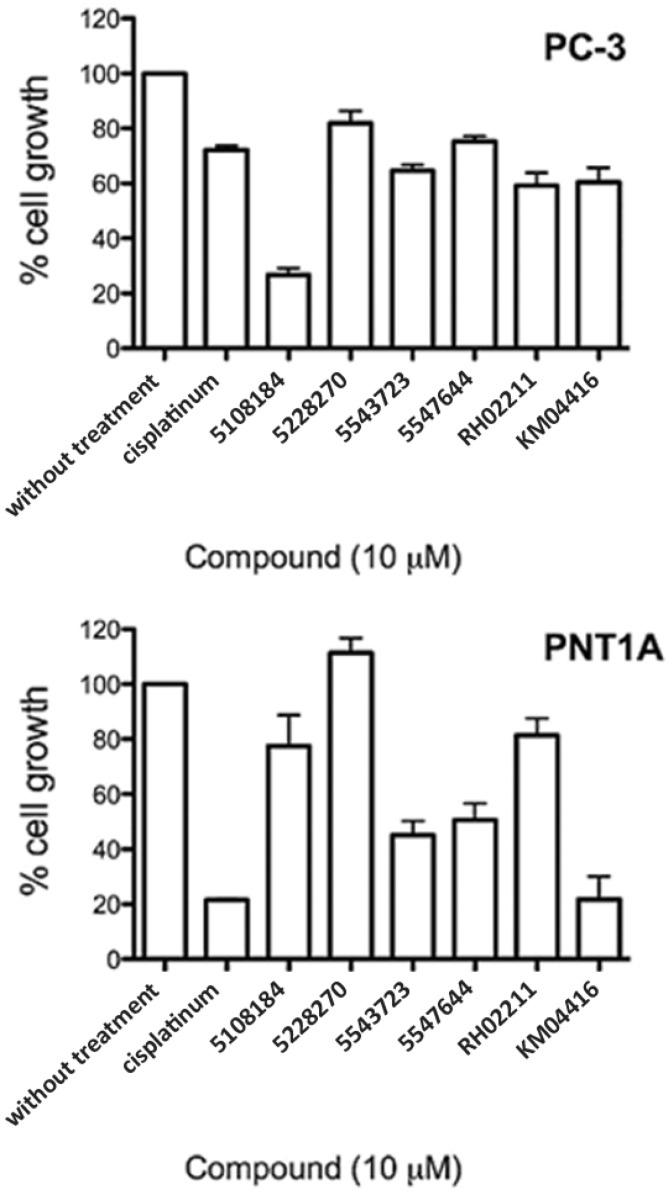
Effect of GPD2 activity inhibitors on the growth of PC-3 and PNT1A cells. Normal and malignant prostate epithelial cell lines were cultured in the presence of 10 µM inhibitors for 72 h. The data are displayed as % cell growth compared to untreated cells. Results represent the average ±SEM of five independent experiments with eight wells per experiment. 10 µM cisplatin was used as a cytotoxic agent positive control. All GPD2 inhibitors and cisplatin inhibited PC-3 cell proliferation significantly (*p* < 0.05, Tukey’s multiple comparison test). Molecules 5477644, 5547644, KM04416 and cisplatin inhibited PNT1A cell proliferation significantly (*p* < 0.05).

The six selected compounds were analyzed for growth inhibition effects in PC-3 cells. Compounds 5108184, RH0211 and KM04416 displayed up to 30% cytotoxicity (Figure 5) as assessed by lactate dehydrogenase release (CytoTox^®^ assay). However, no cytotoxic effects were detected using the Alamar Blue cytotoxicity assay and Trypan Blue cell counts (data not shown).

Initially, the cell-based assay identified cell-permeable oxidative stress effectors. Using sub-libraries with molecules of known properties and modes of action we unveiled peculiarities of the molecular network involved in the ROS generating machinery. Dysregulation of mitochondrial Ca^2+^ homeostasis is recognized to play a key role in several pathologies, including ROS generation. G-protein signaling constitutes an interface between physiological responses and oxidative stress responses. Many small molecule metabolites with messenger functions (dopamine, H5T3, serotonin, glutamate, *etc.*) influence paracrine signaling for redox homeostasis, and affect the level of oxidative stress experienced by prostate cancers.

Among the various compounds identified in this study a number of known herbal antioxidants also emerged. Due to their high tolerability and low toxicity, they were earlier described in several studies as chemopreventative compounds and have received increasing attention due to their anti-initiating and/or anti-promoting effects on tumour growth. Based on chemical uniqueness, we subjected the six most novel substances from this study to further screening and evaluated their ability to affect PC-3 cell growth. Inhibition of GPD2-derived H_2_O_2_ production impaired PC-3 cell growth and survival *in vitro*.

In our analysis, all the tested small molecules emerged as promising compounds with several features underlining their suitability. They significantly inhibited glycerol-3-phosphate oxidoreductase activity, H_2_O_2_ production, and PC-3 cell growth at relatively low concentrations (10 µM). Molecule 5108184 (a benzoxadiazole derivative) inhibited PC-3 cell growth by up to 70% after 3 days of treatment, whereas RH02211 (a benzimidazole derivative), 5477644 (a hydroxybenzaldehyde derivative), and KM04416 (an isothiazolone derivative) inhibited growth by 50%. Compound 5228270 (a methylbenzenesulfonamide derivative) and 5543723 (a benzothiophene derivative) demonstrated up to 30% growth inhibition. They all impacted the pro-oxidative phenotype of PC-3 cells at low concentrations (EC_50_ ranging between 1 and 30 µM). Additionally, 5108184, 5228270, and RH02211 showed no adverse effects on PTN1A cells.

Although the molecules identified in this study display inhibition of glycerophosphate-dependent H_2_O_2_ formation, other modes of action cannot to be excluded at the current stage of our research. Due to their chemical structures, these inhibitors may impair cell function by forming covalent bonds with reactive groups in other biologically important molecules. Fortuitously, derivative analogs of the identified compounds illustrate anti-tumour activity by glutathione S-transferase [38] (benzoxadiazoles) and topoisomerase inhibition [39] (benzimidazoles). The low IC_50_/EC_50_ values make these molecules strong candidates for further investigations of the mechanism of action. Discovering new drug targets for cancer therapy is a significant aim of cancer research.

Early biochemical studies revealed elevated levels of GPD2 activity in fast-growing, undifferentiated tumours, such as hepatomas. In slow-growing, differentiated tumours, GPD2 activity was at normal or slightly reduced levels [40,41,42,43]. GPD1, on the other hand, was reported at a normal level in well-differentiated tumours, and was diminished or absent in fast-proliferating, undifferentiated tumours [40,44,45,46,47,48]. Additionally, the fact that GPD2 is involved in glycolysis/gluconeogenesis and lipolysis/lipid biosynthesis with significant pleiotropic effects on lipid accumulation and intracellular glucose homeostasis [32] underlines the metabolic importance of this enzyme in malignancies. Particular prostate cancers are characterized by a low rate of glycolysis and an increased rate of lipolysis [49]. H_2_O_2_ production from GPD2 may also have molecular implications in cardiovascular disease [50] and atherosclerosis development [51].

Mitochondrial enzymes have been the focus of attention, especially in recent years, as mitochondria play a characteristic role in cell metabolism and cancer development, and specific cancer inhibition may possibly be achieved at this cellular locus [52,53]. Evidence suggests that an excessive increase in ROS levels, often observed in cancer cells, influence various properties of these malignant cells [1]. Therefore, manipulating ROS generation in malignant cells may be a plausible means to control cancer progression [54]. However, the complex relationship between ROS and antioxidant enzyme activity suggests that further characterization of enzyme kinetics will be required. Molecules that limit the oxidative stress of malignancies at the source may deprive tumour cells of ROS-induced growth signaling pathways, and therefore could have the potential to cause growth arrest.

## 4. Conclusions

We have isolated a series of promising molecules that have the potential to lead to the development of novel anti-proliferative agents. In addition to their potential usage as anti-cancer drugs, the small molecules identified here could be developed into valuable tools for analyzing the catalytic mechanisms of GPD2 and probing its ROS-generating mechanism at the molecular level. A thorough functional understanding of GPD2 may lead to drug development in other disease models, like diabetes and atherosclerosis.
